# Intraoperative or postoperative radiotherapy boost in high-risk breast cancer: a matched-pair analysis

**DOI:** 10.1007/s00066-026-02592-3

**Published:** 2026-09-17

**Authors:** Gustavo R. Sarria, Cas Dejonckheere, Jorge Fernandez, Jose Burgos, Adela Heredia, Eleni Gkika, Gustavo J. Sarria, Paola Fuentes-Rivera

**Affiliations:** 1 Department of Radiation Oncology, University Hospital Bonn, University of Bonn, Venusberg Campus 1, Building 55, 53127 Bonn, Germany; 2 Department of Radiotherapy, Instituto Nacional de Enfermedades Neoplasicas, Lima, Peru; 3 Department of Radiation Oncology, Oncosalud-Auna, Lima, Peru

**Keywords:** Intraoperative radiotherapy, Boost, Breast cancer, Early-stage, High-risk

## Abstract

**Purpose:**

The optimal boost technique following breast-conserving surgery for patients with high-risk early breast cancer remains controversial. This study compared oncologic outcomes following an intraoperative radiotherapy (IORT) boost using low-energy X‑rays with those of a conventional external beam radiotherapy (EBRT) boost in a matched-pair cohort.

**Methods:**

Patients treated with breast-conserving surgery followed by either an upfront IORT boost (20 Gy prescribed to the applicator surface) or a postoperative EBRT boost (16 Gy in 8 fractions), sequentially after whole-breast radiotherapy, between 2014 and 2019 were retrospectively identified from a prospectively maintained institutional database. Whole-breast irradiation was delivered after IORT using either conventional or moderately hypofractionated schedules according to institutional practice. The primary endpoint was ipsilateral breast tumour recurrence (IBTR). Secondary endpoints included nodal recurrence, distant metastasis, overall survival (OS), and breast cancer-specific survival (BCSS). Time-to-event outcomes were estimated using the Kaplan-Meier method, whereas treatment comparisons were performed using Cox proportional hazards regression with robust sandwich variance estimates clustered by matched pair.

**Results:**

Following 1:1 matching, 400 patients (200 per group) were included. After a median follow-up of 58 months, the cumulative incidence of local recurrence was 3.5% and 2.0%, respectively, with no statistically significant difference between treatment groups (HR 1.80, 95% CI 0.58–5.58; *p* = 0.312). Likewise, no significant differences were observed for nodal recurrence-free survival (HR 0.98, 95% CI 0.19–5.16; *p* = 0.980), distant metastasis-free survival (HR 0.92, 95% CI 0.38–2.23; *p* = 0.859), breast cancer-specific survival (HR 0.79, 95% CI 0.30–2.10; *p* = 0.634), or overall survival (HR 1.19, 95% CI 0.59–2.40; *p* = 0.636).

**Conclusions:**

In this matched-pair analysis, an intraoperative low-energy X‑ray boost achieved oncologic outcomes comparable to those of a conventional postoperative EBRT boost in patients undergoing breast-conserving therapy for high-risk early breast cancer. These findings support IORT boost as an effective and well-tolerated alternative that may simplify treatment delivery without compromising long-term oncologic outcomes.

## Introduction

Over recent decades, de-escalation strategies have transformed the management of early breast cancer (EBC) [1]. Breast-conserving surgery (BCS) followed by whole-breast irradiation (WBI) demonstrates survival outcomes equivalent or even superior to those of earlier radical surgical approaches [2]. In high-risk patients, the addition of a boost to the tumor bed provides a significant improvement in local control, with up to 60% of patients potentially requiring this approach based on patient and tumor characteristics [3–5]. Several boost delivery techniques have been investigated, most commonly external beam radiotherapy (EBRT) as a sequential boost of 10–16 Gy in 5–8 fractions after WBI, resulting in overall treatment times of 4–7 weeks [6]. More recently, simultaneous integrated boost (SIB) approaches in moderately hypofractionated regimens have been introduced, which shorten treatment duration to 3 weeks [7–9]. These EBRT-based boosts are, however, associated with cosmetic disadvantages and uncertainties in tumor bed localization, particularly in unmarked cavities [10]. A direct intraoperative visualization of the surgical cavity offers the most precise boost targeting. Multicatheter high-dose rate (HDR) brachytherapy is one of the earliest and most extensively studied techniques in this setting, with robust evidence supporting its efficacy [11]. Furthermore, it yields favorable cosmetic outcomes in comparison with EBRT, likely due to reduced skin and subcutaneous tissue exposure [12–14]. Nonetheless, HDR is limited by the need for prolonged hospital stays.

Intraoperative radiotherapy (IORT), delivered either with electrons or low-energy (ie, kilovoltage) X‑rays, has emerged as an alternative technique, enabling immediate irradiation of the tumor bed during surgery [2, 15, 16]. Growing evidence supports its use both as single-fraction accelerated partial breast irradiation (APBI) and as a boost modality in conjunction with postoperative WBI, with potential additional benefits such as improved cosmetic outcomes and an enhanced immune response [17–19]. Herein, we present a matched-pair analysis of sequential EBRT versus upfront IORT boost in patients with high-risk EBC, focussing on disease control and survival outcomes.

## Materials and methods

### Patients and procedures

Patients aged ≥ 18 years with early breast cancer treated at the National Cancer Institute of Peru (Instituto Nacional de Enfermedades Neoplasicas [INEN]) between January 2014 and December 2019 were retrospectively identified from a prospectively maintained institutional intraoperative radiotherapy (IORT) registry. Eligible patients had an Eastern Cooperative Oncology Group (ECOG) performance status of 0–1, unifocal invasive early breast cancer confirmed by mammography and breast ultrasound, with breast magnetic resonance imaging (MRI) performed when clinically indicated, and underwent breast-conserving surgery (BCS) followed by whole-breast irradiation (WBI). Patients received either an IORT boost or a postoperative external beam radiotherapy (EBRT) boost. Allocation was primarily determined by institutional logistics and operating room availability, as all patients had initially been screened for IORT.

All patients presented at least one established indication for tumour-bed boost irradiation, namely age ≤ 50 years, pathological tumour stage ≥ pT2 (according to the 7th edition of the AJCC Cancer Staging Manual), grade 3 disease, triple-negative breast cancer, or HER2-positive disease.

Exclusion criteria comprised metastatic disease at diagnosis, multifocal or multicentric breast cancer, previous ipsilateral breast irradiation, mastectomy, neoadjuvant radiotherapy, incomplete clinicopathological or follow-up data precluding outcome assessment, failure to complete postoperative whole-breast irradiation, or failure to fulfil institutional criteria for breast-conserving therapy.

Initial staging consisted of bilateral mammography and breast ultrasound in all patients. Breast MRI, computed tomography, bone scintigraphy, or other systemic staging examinations were performed when clinically indicated according to institutional practice during the study period.

Clinically node-negative patients underwent sentinel lymph node biopsy. Patients with histologically confirmed nodal involvement underwent axillary lymph node dissection according to institutional guidelines applicable during the study period. Regional nodal irradiation was delivered following multidisciplinary tumour board recommendations based on pathological nodal burden and additional clinicopathological risk factors.

Hormone receptor positivity was defined as ≥ 1% nuclear staining according to the ASCO/CAP recommendations. Adjuvant chemotherapy, when indicated, was completed before initiation of postoperative radiotherapy.

Whole-breast irradiation was delivered using either conventional fractionation (45–50 Gy in 1.8–2.0 Gy per fraction) or moderately hypofractionated schedules (40.05 Gy in 15 fractions or equivalent institutional schedules) using three-dimensional conformal radiotherapy (3D-CRT), intensity-modulated radiotherapy (IMRT), or volumetric-modulated arc therapy (VMAT).

The EBRT boost consisted of 16 Gy delivered in eight fractions. IORT was delivered immediately after tumour excision using the 50-kV INTRABEAM® system (Carl Zeiss Meditec AG, Oberkochen, Germany). A spherical applicator was selected intraoperatively to ensure optimal contact with the surgical cavity. The median applicator diameter was 3.5 cm (range, 2.0–5.0 cm). The prescribed dose was 20 Gy to the applicator surface, corresponding to approximately 30% of the prescribed dose at a depth of 1 cm. Lower doses were permitted at the discretion of the treating radiation oncologist when anatomical constraints, such as a skin-to-cavity distance < 1 cm, were present.

Patients were followed according to institutional protocols every six months during the first two years and annually thereafter. Follow-up consisted of clinical examination, annual mammography, and additional imaging, including ultrasound or breast MRI, when clinically indicated.

### Endpoints

The primary endpoint was ipsilateral breast tumour recurrence (IBTR), defined as histologically or radiologically confirmed recurrence within the ipsilateral breast. Secondary endpoints included nodal recurrence, distant metastasis, overall survival (OS), and breast cancer-specific survival (BCSS).

Time-to-event endpoints comprised cumulative local recurrence (LR), nodal recurrence-free survival (NRFS), distant metastasis-free survival (DMFS), OS, and BCSS. For LR, NRFS, and DMFS, patients experiencing the corresponding event were considered to have reached the endpoint, whereas all remaining patients were censored at the date of last clinical follow-up. OS was calculated from surgery to death from any cause, whereas BCSS considered only deaths attributable to breast cancer as events.

### Statistical analysis

Baseline demographic, tumour, and treatment characteristics were summarised using descriptive statistics. Continuous variables are presented as mean ± standard deviation (SD) or median with interquartile range (IQR), as appropriate. Categorical variables are presented as frequencies and percentages.

Because the study employed a matched-pair design, all comparative analyses accounted for within-pair dependence. Continuous paired variables were analysed using paired Student’s t‑tests or Wilcoxon signed-rank tests, as appropriate. Paired categorical variables were analysed using McNemar’s test.

Time-to-event outcomes (LR, NRFS, DMFS, OS, and BCSS) were estimated using the Kaplan-Meier method. Comparisons between treatment groups were performed using Cox proportional hazards regression with robust sandwich variance estimates clustered by matched pair. Hazard ratios (HRs), 95% confidence intervals (CIs), and corresponding *p*-values were obtained from these clustered Cox models.

All statistical tests were two-sided, and *p* values < 0.05 were considered statistically significant. Statistical analyses were performed using R (R Foundation for Statistical Computing, Vienna, Austria) with the survival (Therneau T (2026). A Package for Survival Analysis in R. R package version 3.8‑6, https://CRAN.R-project.org/package=survival.) and survminer (Kassambara A, Kosinski M, Biecek P (2026). survminer: Drawing Survival Curves using ‘ggplot2’. https://doi.org/10.32614/CRAN.package.survminer, R package version 0.5.2, https://CRAN.R-project.org/package=survminer.) packages.

## Results

A total of 1178 patients were screened for eligibility, of whom 564 were enrolled in the institutional IORT registry. After application of the predefined eligibility criteria and 1:1 matched-pair selection, 400 patients (200 per treatment group) were included in the final analysis.

Baseline patient, tumour, and treatment characteristics are summarised in Table 1. The median age at diagnosis was 51 years in both treatment groups (mean ± SD: 52.7 ± 10.4 years for IORT and 52.7 ± 11.9 years for EBRT; *p* = 0.963). Clinical tumour stage, nodal stage, histological subtype, systemic treatment, and hormone receptor status were well balanced between groups. However, several clinicopathological differences remained after matching. Patients treated with IORT more frequently presented with associated ductal carcinoma in situ (41.5% vs. 22.5%; *p* < 0.001), closer in-situ surgical margins (≤ 2 mm; *p* < 0.001), and higher Ki-67 proliferation indices (median 25% vs. 20%; *p* = 0.004). Conversely, patients in the EBRT cohort more frequently received three-dimensional conformal radiotherapy and moderately hypofractionated whole-breast irradiation (both *p* < 0.001).

**Table 1:** Baseline patient and tumour characteristics

	IORT (*n* = 200)	EBRT (*n* = 200)	p
*Age, years*			
Median [range]	51 [27-86]	51 [26-85]	0.993
*Side*			
Right	97 (48.5%)	92 (46.0%)	–
Left	103 (51.5%)	108 (54.0%)	0.699
*cT*			
cT1a	20 (10.0%)	14 (7.0%)	–
cT1b	29 (14.5%)	26 (13.0%)	
cT1c	53 (26.5%)	53 (26.5%)	
cT 2	71 (35.5%)	84 (42.0%)	
cT 3	27 (13.5%)	23 (11.5%)	0.636
*cN*			
cN 0	160 (80.0%)	152 (76.0%)	–
cN 1	40 (20.0%)	48 (24.5%)	0.754
*Neoadjuvant chemotherapy*			
Yes	63 (31.5%)	57 (28.5%)	–
No	137 (68.5%)	143 (71.5%)	0.801
*Antihormonal therapy*			
Yes	152 (76.0%)	154 (77.0%)	–
No	44 (22.0%)	46 (23.0%)	1.000
Not registered	4	0	–
*Adjuvant chemotherapy*			
Yes	151 (75.5%)	150 (75.0%)	–
No	49 (24.5%)	50 (25.0%)	1.000
*Histology*			
IDC/NST	179 (89.5%)	177 (88.5%)	–
Other	21 (10.5%)	23 (11.5%)	0.951
*Tumor size, mm*			
Median [IQR]	18 [11-23]	20 [15-25]	0.23
*Applicator diameter, cm*	3.5 (2.0–5.0)	–	
*pT*			
ypT 0	23 (11.5%)	18 (9.0%)	–
ypT1a	15 (7.5%)	13 (6.5%)	
ypT1b	23 (11.5%)	24 (12.0%)	
ypT1c	68 (34.0%)	64 (32.0%)	
ypT 2	68 (34.0%)	79 (39.5%)	
ypT 3	0 (0.0%)	2 (1.0%)	0.387
*pN*			
ypN 0	140 (70.0%)	141 (70.5%)	–
ypN 1	46 (23.0%)	54 (27.0%)	
ypN 2	12 (6.0%)	4 (2.0%)	
ypN 3	2 (1.0%)	0 (0.0%)	0.561
Not registered	0	1	–
*Grade*			
G1	15 (7.5%)	21 (10.5%)	–
G2	109 (54.5%)	120 (60.0%)	
G3	68 (34.0%)	59 (29.5%)	0.223
Not registered	8	0	–
*Lymphovascular invasion*			
Yes	40 (20.0%)	41 (20.5%)	–
No	150 (75.0%)	153 (76.5%)	0.828
Not registered	10	6	–
*Additional DCIS*			
Yes	83 (41.5%)	45 (22.5%)	–
No	117 (58.5%)	151 (75.5%)	<0.001
Not registered	0	4	–
*Margins from in-situ component*			
≤2 mm	25 (30.1%)	8 (17.8%)	–
>2 mm	11 (13.3%)	2 (4.4%)	<0.001
Not registered *	41	35	–
*Estrogen receptors*			
Positive	150 (75.0%)	153 (76.5%)	–
Negative	46 (23.0%)	47 (23.5%)	0.907
Not registered	4	0	–
*Progesterone receptors*			
Positive	133 (66.5%)	136 (68.0%)	–
Negative	63 (31.5%)	64 (32.0%)	1.000
Not registered	4	0	–
*HER 2*			
Positive	45 (22.5%)	50 (25.0%)	–
Negative	147 (73.5%)	149 (74.5%)	0.649
Not registered	8	1	–
*Ki-67, %*			
Median [range]	25 [5-90]	20 [1-90]	0.004
Not registered, *n*	10	0	–
*Radiotherapy technique*			
3D-CRT	43 (21.5%)	134 (67.0%)	–
IMRT	71 (35.5%)	2 (1.0%)	
VMAT	86 (43.0%)	64 (32.0%)	<0.001
*Radiotherapy regime*			
Conventional fractionation (1.8–2 Gy/fx)	83 (41.5%)	47 (23.5%)	–
Hypofractionation (> 2 Gy/fx)	116 (58.0%)	153 (76.5%)	<0.001
Not registered	1	0	–

*IORT* intraoperative radiotherapy, *EBRT* external body radiotherapy, *3D-CRT* three-dimensional conformal radiation therapy, *IMRT* intensity-modulated radiation therapy, *VMAT* volumetric-modulated arc therapy, *Gy* Gray, *fx* fraction, *IDC* invasive ductal carcinoma, *NST* non-specific type, *IQR* interquartile range, *DCIS* ductal carcinoma in-situ, *HER2* human epidermal growth factor receptor 2, *Ki-67* proliferation index

*From the group of patients with DCIS

The median follow-up was 58.2 months (range, 3.0–108.0 months) for patients treated with IORT and 58.5 months (range, 2.0–123.0 months) for those treated with EBRT. Among patients receiving IORT, the median interval between surgery and initiation of postoperative whole-breast irradiation was 154 days (interquartile range, 84–286 days).

**Fig. 1:**
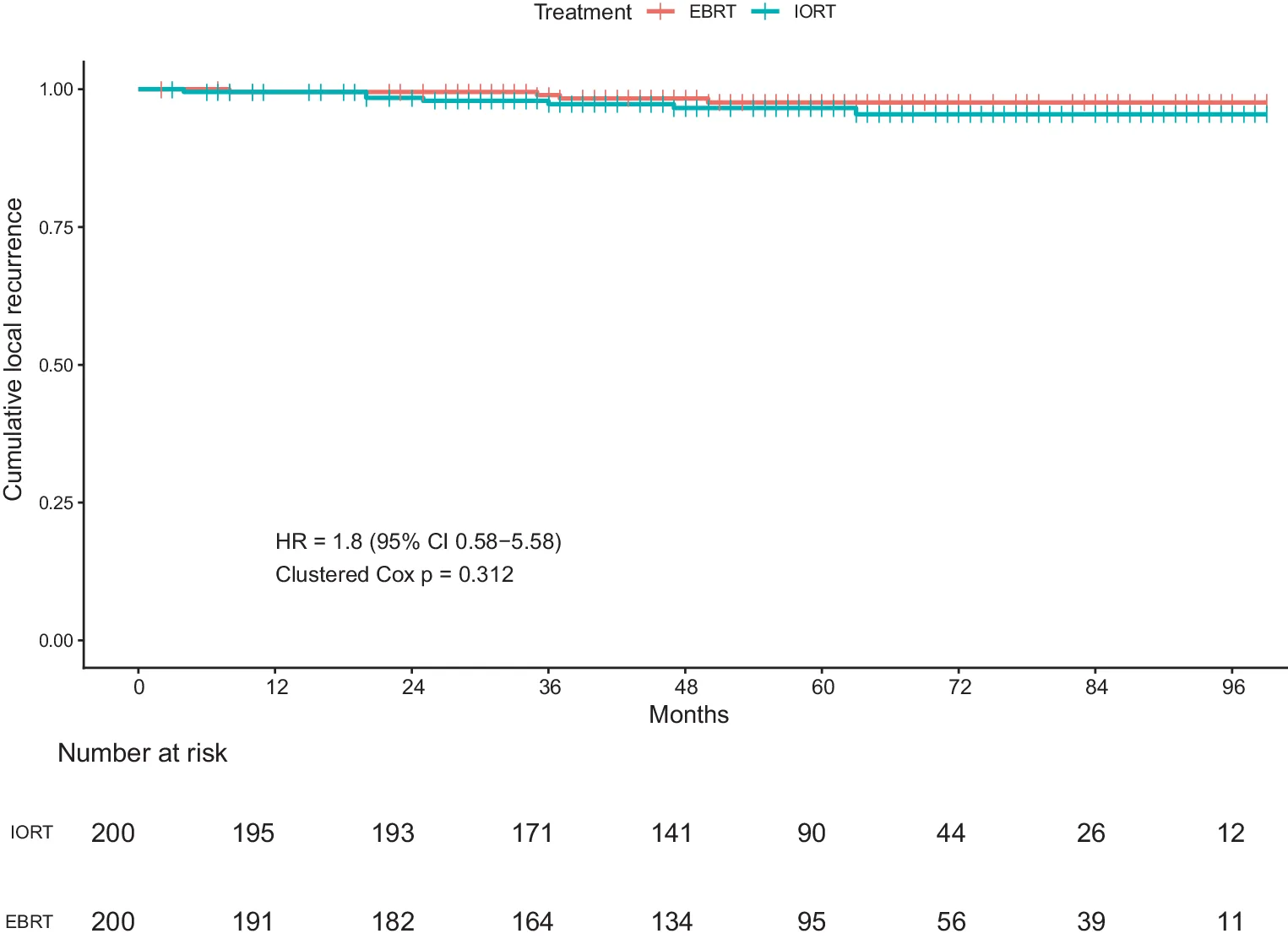
Cumulative local recurrence represented by Kaplan-Meier estimates. (*IORT* intraoperative radiotherapy, *EBRT* external body radiotherapy)

Observed oncologic events are summarised in Table 2. Ipsilateral breast tumour recurrence (IBTR—LR) occurred in 3.5% following IORT and 4 patients (2.0%) following EBRT. No statistically significant difference was observed between treatment groups (HR 1.80, 95% CI 0.58–5.58; *p* = 0.312; Fig. 1).

**Table 2:** Treatment outcomes

	IORT (*n* = 200)	EBRT (*n* = 200)	*p*
*Local recurrence*			
Yes	7 (3.5%)	4 (2.0%)	–
No	193 (96.5%)	196 (98.0%)	0.505
*Nodal recurrence*			
Yes	3 (1.5%)	3 (1.5%)	–
No	197 (98.5%)	197 (98.5%)	1.000
*Distant metastases*			
Yes	9 (4.5%)	10 (5.0%)	–
No	191 (95.5%)	190 (95.0%)	1.000
*Status*			
Deceased	17 (8.5%)	15 (7.5%)	–
Alive	183 (91.5%)	185 (92.5%)	0.850
*Cause-specific survival*			
Cancer-related death	7 (3.5%)	10 (5.0%)	–
Other causes	10 (5.0%)	5 (2.5%)	
Alive	183 (91.5%)	185 (92.5%)	0.343

*IORT* intraoperative radiotherapy, *EBRT* external body radiotherapy

**Fig. 2:**
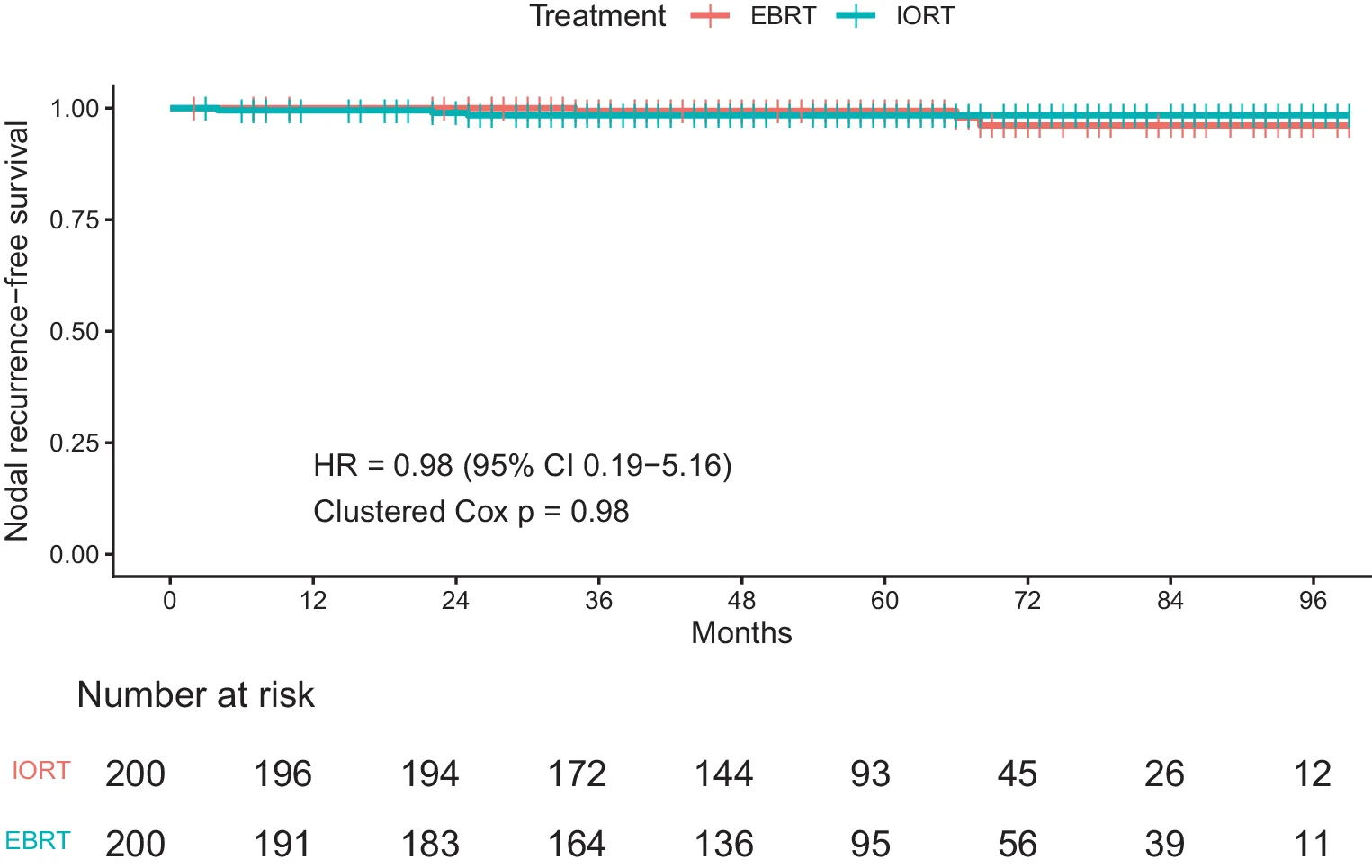
Nodal recurrence-free survival represented by Kaplan-Meier estimates. (*IORT* intraoperative radiotherapy, *EBRT* external body radiotherapy)

Nodal recurrence occurred in three patients (1.5%) in each treatment group. The estimated 5‑year cumulative incidence of nodal recurrence was 1.6% following IORT and 0.6% following EBRT. Nodal recurrence-free survival was comparable between groups (HR 0.98, 95% CI 0.19–5.16; *p* = 0.980; Fig. 2).

**Fig. 3:**
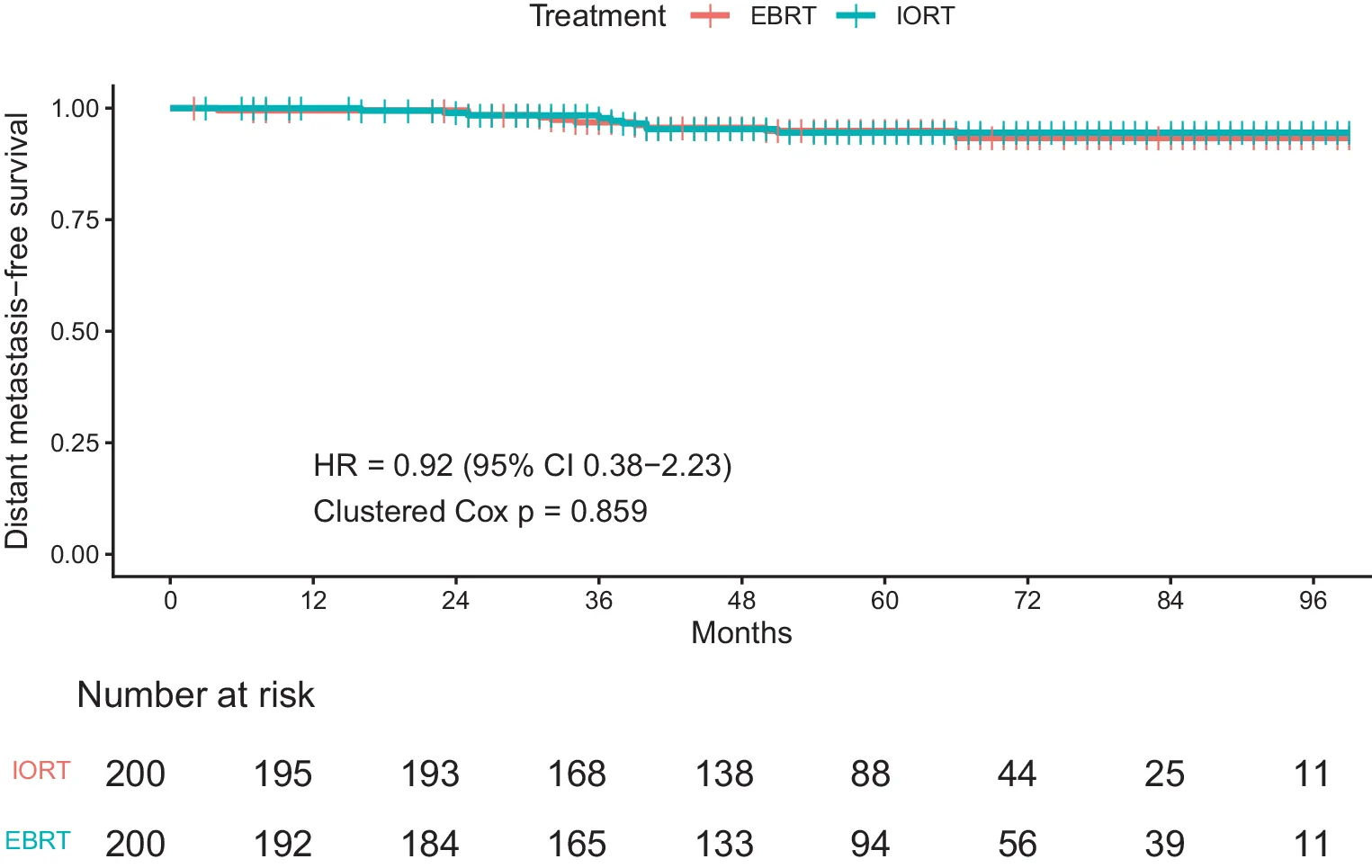
Distant metastases-free survival represented by Kaplan-Meier estimates. (*IORT* intraoperative radiotherapy, *EBRT* external body radiotherapy)

Distant metastases developed in 9 patients (4.5%) treated with IORT and 10 patients (5.0%) treated with EBRT. The estimated 5‑year cumulative incidences were 5.5% and 5.1%, respectively. No statistically significant difference in distant metastasis-free survival was observed (HR 0.92, 95% CI 0.38–2.23; *p* = 0.859; Fig. 3).

**Fig. 4:**
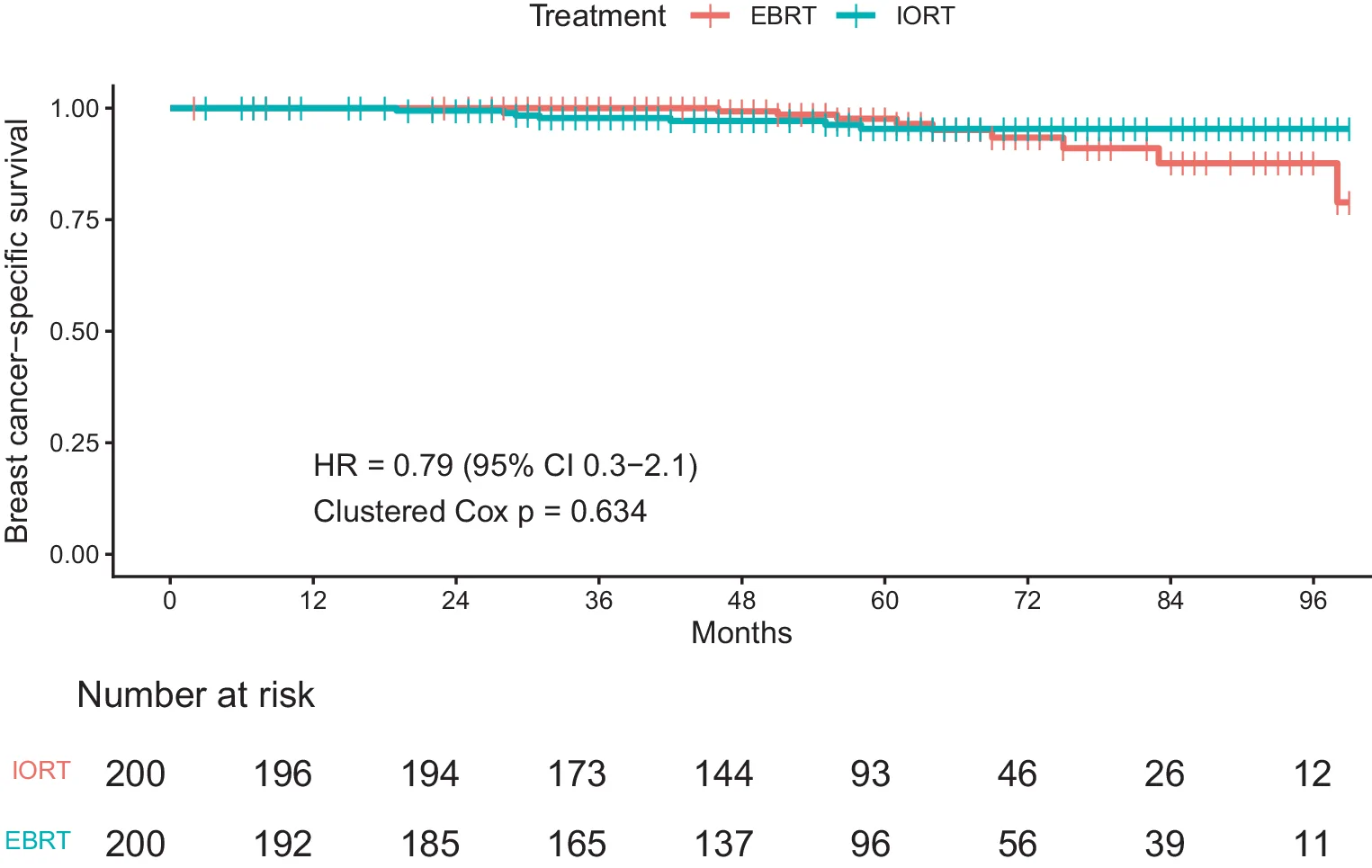
Overall survival represented by Kaplan-Meier estimates. (*IORT* intraoperative radiotherapy, *EBRT* external body radiotherapy)

**Fig. 5:**
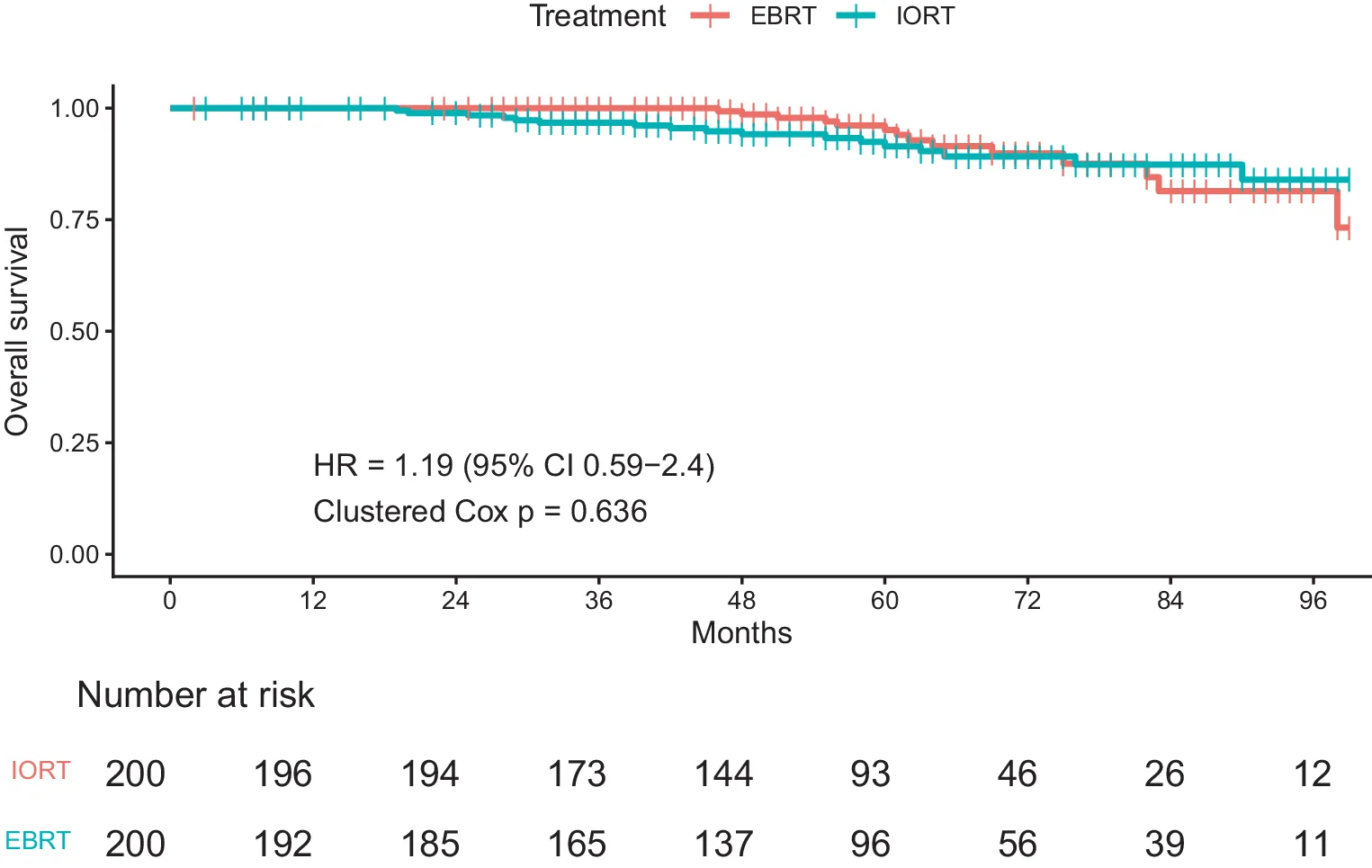
Breast cancer-specific survival represented by Kaplan-Meier estimates. (*IORT* intraoperative radiotherapy, *EBRT* external body radiotherapy)

Breast cancer-specific survival and overall survival were likewise comparable between treatment groups. Estimated 5‑year breast cancer-specific survival was 95.4% and 98.6%, respectively (HR 0.79, 95% CI 0.30–2.10; *p* = 0.634; Fig. 4). The estimated 5‑year overall survival was 91.5% following IORT and 98.0% following EBRT (HR 1.19, 95% CI 0.59–2.40; *p* = 0.636; Fig. 5).

## Discussion

In this matched-pair analysis of patients with high-risk early breast cancer undergoing breast-conserving therapy, an intraoperative low-energy X‑ray boost achieved oncologic outcomes comparable to those of a conventional postoperative EBRT boost. No statistically significant differences were observed in local recurrence-free survival, nodal recurrence-free survival, distant metastasis-free survival, breast cancer-specific survival, or overall survival.

Despite the absence of statistically significant differences, the present findings should be interpreted within the context of the clinicopathological characteristics of the study population. Patients treated with IORT presented a higher prevalence of adverse pathological features, including associated ductal carcinoma in situ, closer in-situ surgical margins, and higher Ki-67 expression, all of which have traditionally been associated with an increased risk of local recurrence. Nevertheless, local control remained comparable between treatment groups, suggesting that an upfront IORT boost provides effective tumour-bed dose intensification even in patients with less favourable pathological characteristics.

Our findings are consistent with previously published studies evaluating IORT boost strategies. Wenz et al. first demonstrated the feasibility and encouraging local control associated with low-kilovoltage IORT boost during breast-conserving surgery, whereas Stoian et al. subsequently reported comparable oncologic outcomes between IORT boost and simultaneously integrated boost approaches [23, 24]. Likewise, the recently published TARGIT-BQR registry demonstrated excellent long-term local control and favourable toxicity following planned IORT boost combined with postoperative WBI, further supporting the role of IORT as an effective boost technique in appropriately selected patients [13, 14, 21]. Our matched-pair analysis extends these observations by directly comparing IORT with a conventional postoperative EBRT boost while accounting for baseline differences between treatment groups.

Several factors may explain the comparable oncologic outcomes observed. Unlike postoperative EBRT boost, IORT enables direct visualisation of the surgical cavity, thereby minimising uncertainties associated with postoperative anatomical changes, seroma formation, clip migration, or oncoplastic reconstruction [10]. Accurate dose delivery to the tumour bed at the time of surgery may reduce the risk of geographical miss while simultaneously avoiding treatment prolongation associated with sequential EBRT boost. Furthermore, delivery of 20 Gy to the applicator surface results in a high biologically effective dose to the tissue at greatest risk of harbouring microscopic residual disease, which may partially compensate for the less favourable pathological characteristics observed in the IORT cohort [16, 18].

The increasing adoption of moderately hypofractionated WBI and simultaneously integrated boost techniques has substantially shortened postoperative radiotherapy schedules [7–9]. Nevertheless, sequential EBRT boost remains widely used in many institutions, and access to advanced planning techniques is not universal. Consequently, IORT continues to represent an attractive alternative, particularly in centres seeking to simplify treatment delivery, reduce hospital visits, and optimise utilisation of radiotherapy resources while maintaining excellent local control. These practical advantages may be particularly relevant in low- and middle-income countries and in healthcare systems with limited radiotherapy capacity.

An important strength of the present study is the matched-pair design, which reduced baseline imbalances between treatment groups while reflecting routine clinical practice. Nonetheless, several limitations should be acknowledged. First, this was a retrospective single-centre study and therefore remains susceptible to residual confounding despite matching. Second, the relatively small number of oncologic events resulted in wide confidence intervals, limiting the precision of hazard ratio estimates. Third, toxicity, cosmetic outcomes, and patient-reported outcomes were not systematically collected in both treatment groups and therefore could not be compared comprehensively [26]. Finally, allocation to IORT was influenced primarily by institutional logistics rather than randomisation, introducing the possibility of unmeasured selection bias despite the matched design.

The ongoing international TARGIT‑B trial is expected to provide the highest level of evidence regarding the comparative effectiveness of IORT and EBRT boost strategies by prospectively evaluating oncologic outcomes together with toxicity, cosmesis, patient-reported outcomes, and cost-effectiveness. Until those results become available, the present study provides additional real-world evidence supporting the use of low-energy X‑ray IORT boost as an effective and clinically practical alternative to conventional postoperative EBRT boost in appropriately selected patients with high-risk early breast cancer.

## Conclusion

This analysis suggests that both upfront IORT and sequential EBRT boosts achieve comparable local disease control rates in patients with high-risk EBC. In settings where sequential or integrated EBRT boost is technically challenging, resource-intensive, or unavailable, IORT represents a valuable alternative that shortens treatment duration and simplifies boost delivery.

## Data Availability

All data supporting the findings of this work are included in the article.
